# MSRS-DETR: End-to-End Object Detection for Multi-Scale Remote Sensing

**DOI:** 10.3390/s25185734

**Published:** 2025-09-14

**Authors:** Jie Yuan, Shuyi Feng, Hao Han

**Affiliations:** 1 College of Computer Science and Technology, Nanjing University of Aeronautics and Astronautics, Nanjing 210024, China; yuanjie_6952@163.com (J.Y.); feng_shu_yi@aliyun.com (S.F.); 2 Shanghai Aerospace Electronic and Communication Equipment Research Institute, Shanghai 201109, China

**Keywords:** remote sensing, object detection, DETR, frequency–spatial fusion, frequency-domain analysis, small object detection, multi-scale detection

## Abstract

Remote sensing imagery (RSI) object detection is critical to many applications, yet mainstream detectors analyse only spatial features and, because of spectral bias, fail to learn high-frequency information adequately, resulting in performance bottlenecks under cluttered backgrounds, distractors, and multi-scale targets, especially small ones. To break these limitations, we propose MSRS-DETR, an end-to-end framework that deeply fuses spatial and frequency cues. The approach introduces three key innovations: (1) C2f_FAT_NET, a frequency-attention-enhanced lightweight residual backbone that provides richer dual-domain features with fewer parameters; (2) an Entanglement Transformer Block (ETB) in the encoder that refines deep semantics via cross-domain frequency–spatial interaction and suppresses background interference; and (3) S2-CCFF, a shallow-feature-extended bidirectional fusion path that markedly improves the retention and utilisation of fine details for small objects. Experiments on HRSC2016 and ShipRSImageNet demonstrate the effectiveness and generalisation of this spatial–frequency paradigm: relative to the baseline, MSRS-DETR reduces parameters by 29.1%, boosts inference speed by 12.4% and 8.4%, and raises mAP_50-95_ by 1.69% and 2.16%, respectively.

## 1. Introduction

Object detection is a cornerstone of computer vision, and its historical trajectory closely follows the evolution of deep learning paradigms. The field has progressed from early detectors that relied on handcrafted features through convolutional neural network (CNN)-based architectures to the recent emergence of Transformer-based models, steadily improving in accuracy, speed, and generalisation. Within this context, remote sensing object detection (RSOD) supports vital tasks such as urban planning, resource exploration, environmental monitoring, and national security, and it therefore continues to attract significant research and practical attention [1].

When state-of-the-art detectors designed for natural images are applied directly to remote sensing imagery (RSI), their performance is often constrained by domain-specific factors, including sensor noise, cluttered backgrounds, high semantic similarity between targets and surroundings, extreme scale variation, and the high density of small objects.

Mainstream CNN and Transformer detectors learn and represent features solely in the spatial domain, overlooking the information embedded in the frequency domain. According to Fourier theory, low-frequency components encode global contours and smooth regions, whereas high-frequency components capture edges and textures [2]. Small objects in RSI correspond mainly to high-frequency signals, and cluttered backgrounds exhibit distinctive frequency-domain patterns. Moreover, deep networks show a spectral bias [3]; they fit low-frequency information first, which hinders the modelling of high-frequency details. A detector that seamlessly fuses spatial and frequency cues is therefore likely to overcome these intrinsic limitations and advance high-precision RSOD.

Motivated by this insight, and taking the real-time end-to-end detector RT-DETR [4] as our baseline, we develop MSRS-DETR, a multi-scale detector tailored for RSI.

The main contributions of this work are as follows:

1. An efficient end-to-end framework for RSOD that integrates frequency-domain analysis into the backbone, encoder, and feature-fusion stages, thereby improving multi-scale detection in complex scenes.
2. C2f_FAT_NET, a lightweight residual backbone equipped with frequency attention, which reduces parameters while enhancing the discriminative power of extracted features.
3. An Entanglement Transformer Block (ETB) that performs joint frequency–spatial attention to distil robust high-level semantics and suppress background interference.
4. S2-CCFF, a shallow-feature-augmented bidirectional fusion addition that preserves high-resolution detail and boosts the recall of small targets while introducing modest latency overhead along the fusion path.

The remainder of this paper is organised as follows. Section 2 reviews related work. Section 3 details the proposed MSRS-DETR, including a lightweight backbone refinement that augments the C2f/RepC3 blocks to better preserve fine-grained details, the Entanglement Transformer Block (ETB) for jointly modelling frequency and spatial cues, and the S2-CCFF bidirectional fusion across scales. Section 4 briefly summarises the experimental setup and datasets and reports the main quantitative results with concise qualitative examples. Section 5 concludes this paper and discusses limitations and future directions.

## 2. Related Work

### 2.1. CNN-Based Remote Sensing Object Detection

Since the rise of deep learning, CNN-driven algorithms have rapidly dominated object detection and have evolved along two main trajectories. The first comprises two-stage detectors typified by the R-CNN family [5,6,7], which follow a proposal-then-refinement paradigm. By generating high-quality candidate boxes through a region proposal network, these methods achieve strong accuracy, though their multi-stage pipelines restrict real-time deployment. The second trajectory favours speed and is represented by one-stage detectors such as the You Only Look Once (YOLO) series [8]. By eliminating the proposal stage and casting detection as direct regression and classification, YOLO has, through successive iterations, struck an exceptional balance between speed and precision. The latest version, YOLOv8 [9], introduces a more efficient C2f backbone, an anchor-free detection head, and advanced loss functions, making it a widely adopted baseline in both academia and industry. To tackle the intrinsic multi-scale challenge of CNNs, Feature Pyramid Networks (FPNs) [10] add a top-down pathway with lateral connections, merging deep semantic information with shallow spatial detail and becoming a standard component of modern detectors.

When these advanced CNN detectors are applied directly to remote sensing imagery, their inherent limitations become evident. The local receptive field of convolutions hampers the capture of the global context and long-range dependencies required for large-scene interpretation. Repeated down-sampling, essential for building deep semantic features, inevitably discards information; the faint signatures of small objects, common in RSI, are easily overwhelmed during propagation. Consequently, the upper bound of CNN performance in complex remote sensing scenarios is constrained by the architecture itself, motivating research into paradigms with stronger global modelling capacity.

In recent years, many remote sensing detectors have extended fast YOLO-style baselines to address domain challenges, such as small, sparse targets and cluttered backgrounds, while keeping real-time efficiency. Representative examples include MSA-YOLO, which introduces multi-scale strip attention, GS-PANet fusion, and a Wise-Focal CIoU loss to suppress background noise and balance sample contributions [11]; YOLO-RS, which couples ASFF/OSPP-like aggregation with a lightweight head to improve accuracy and throughput on aerial benchmarks [12]; and YOLO-RSA, a multi-scale design specialised for ship scenarios in optical imagery [13]. Under adverse weather, CM-YOLO further applies component-decoupling background suppression and local–global semantic mining to maintain robustness in cloud/mist scenes [14]. Complementary surveys synthesise that attention modules, lightweight backbones, and enhanced multi-scale fusion (e.g., ASFF/BiFPN variants) are effective methods for improving detection in RSI without sacrificing speed [15,16].

### 2.2. Transformer-Based Remote Sensing Object Detection

To overcome the locality of CNNs, the Transformer architecture, originally devised for natural language processing, has been introduced into computer vision and is now reshaping object detection. Starting with the Vision Transformer (ViT) [17], its self-attention mechanism enables direct interactions between arbitrary pixels, yielding a true global receptive field.

DETR (Detection Transformer) [18] is the seminal work in this line. By adopting an encoder–decoder Transformer and learnable object queries, DETR reformulates detection as an end-to-end set prediction task, discarding anchors and non-maximum suppression in favour of bipartite matching and set loss supervision. Despite its elegance, the first version converges slowly and struggles with small objects. Deformable DETR [19] addresses these issues by introducing deformable attention, which focuses computation on a sparse set of sampling points, greatly reducing complexity, accelerating convergence, and improving small-object recall. To extend DETR’s end-to-end advantages to real-time scenarios, RT-DETR [4] employs a lightweight hybrid encoder comprising a single Transformer layer on top of deep CNN features and adopts uncertainty-minimal query selection. It thus achieves very high throughput while maintaining accuracy, performing competitively with, and in many cases surpassing, heavily optimised YOLO variants on the reported benchmarks, and establishing a strong standard for real-time end-to-end detection.

Although Transformer-based detectors excel at global modelling, several limitations remain in remote sensing. First, they typically require large-scale pre-training, whereas well-annotated RSI datasets are scarce. Second, in hybrid designs such as RT-DETR, image features still undergo multiple CNN down-sampling operations before entering the Transformer, so the loss of small-object information persists [20], leaving the subsequent attention layers with insufficient detail.

Building on the real-time encoder–decoder paradigm, RS-DETR adapts RT-DETR to aerial scenarios via cross-scale gating, EBiFPN-style fusion, and refined IoU objectives, reporting improved accuracy while preserving throughput on representative benchmarks [21]. Recent surveys further emphasise that to stabilise optimisation and accommodate diverse object scales in remote sensing, Transformer components are increasingly combined with CNN pyramids and domain-tailored fusion necks [16]. Overall, these advances suggest that carefully designed fusion and loss formulations are key to transferring Transformer-style global reasoning to remote sensing imagery.

## 3. Methodology

**Figure 1:**
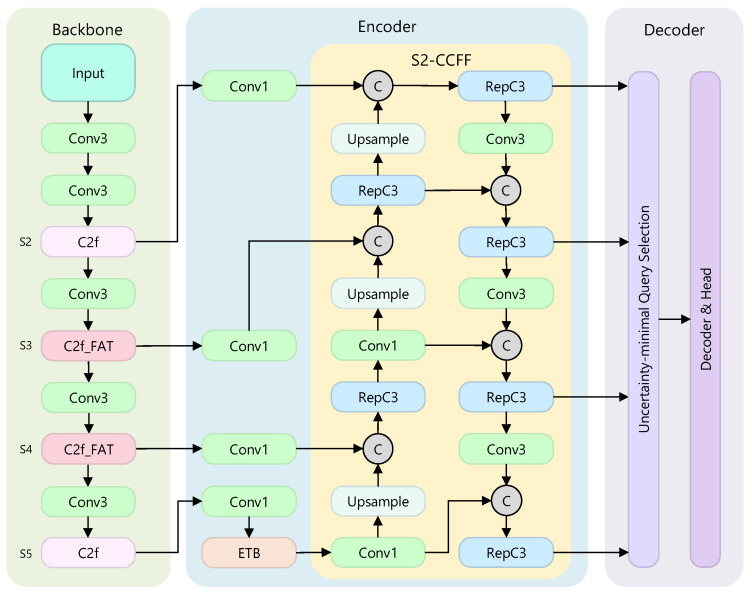
Overall architecture of MSRS-DETR.

To address the widespread challenges in remote sensing imagery of detecting multi-scale objects, especially small targets, coping with heavy background clutter, and overcoming the limited representational capacity of existing end-to-end detectors, we build on the high-performance real-time detector RT-DETR [4] and propose MSRS-DETR, a new multi-scale remote sensing detection model that revises the backbone, encoder, and decoder designs. Figure 1 gives an overview of the architecture, which comprises a lightweight frequency-aware residual backbone C2f_FAT_NET, an Entanglement Transformer Block (ETB) for frequency–spatial feature extraction, and an S2-CCFF fusion module that leverages shallow features.

### 3.1. Backbone

The backbone underpins the detector, and the quality of its features directly sets the performance ceiling. An effective backbone should extract discriminative multi-scale features while keeping computational cost low. In MSRS-DETR we introduce C2f_FAT_NET, a frequency-aware backbone illustrated in Figure 1. Classic C2f blocks handle the shallow S2 and deepest S5 stages for generic features, whereas the middle stages S3 and S4 adopt the new C2f_FAT_ blocks for stronger discrimination.

The C2f block, derived from CSPNet [22], splits the input along channels into a shortcut branch and a bottleneck branch, then concatenates and fuses them with a 1×1 convolution, thus enriching gradients and reducing redundancy. However, its bottleneck relies solely on standard convolutions, a spatially local operation that lacks long-range context.

**Figure 2:**
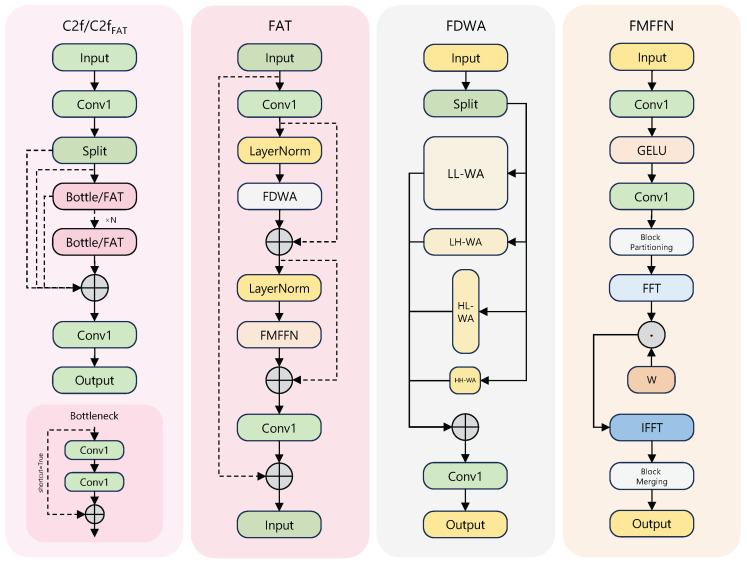
Structures of the C2f block and the proposed C2f_FAT_ block.

We therefore replace the bottleneck with a Frequency-Aware Transformer (FAT) module [23], which extends analysis from the spatial domain to the joint frequency–spatial domain. As shown in Figure 2, FAT contains Frequency-Decomposition Window Attention (FDWA) and a Frequency-Modulation Feed-Forward Network (FMFFN).

FDWA runs four attention windows of different shapes in parallel: a large square window for low-frequency context (LL-WA), a small square window for high-frequency detail (HH-WA), and two rectangular windows for vertical (HL-WA) and horizontal (LH-WA) anisotropic features. Thus, a single block can decompose multi-scale and multi-directional frequency components that describe oriented ships or textured vegetation.

FMFFN adaptively refines the features as

(1) $$X_{\mathrm{out}}=B^{-1}\left[F^{-1}\left(F\left[B\left(X_{\mathrm{ffn}}\right)\right]⊙W\right)\right]$$

where $X_{\mathrm{ffn}}$ is the input of the block, B(·) and $B^{-1}\left(\cdot \right)$ denote block partition and merge, and F, $F^{-1}$ are the fast Fourier transform and its inverse. The learnable filter *W* modulates the spectrum, amplifying informative frequencies and suppressing noise.

By integrating FAT, the C2f_FAT_ block evolves from a purely spatial extractor into a dual-domain module. Despite higher internal complexity than a single bottleneck, its stronger representation allows fewer parameters than stacking multiple convolutions and furnishes richer cues for downstream detection, particularly where pure spatial features fall short.

### 3.2. Encoder

#### 3.2.1. Entanglement Transformer Block

**Figure 3:**
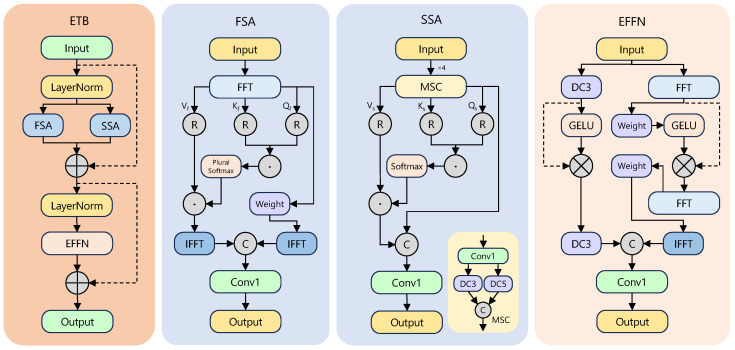
Architecture of the ETB module.

To extract robust features when targets and background share high visual similarity, we append an Entanglement Transformer Block (ETB) [24] after the deepest backbone stage S5. ETB jointly models features in frequency and spatial domains through parallel processing and deep interaction. Figure 3 outlines its three parts: frequency self-attention (FSA), spatial self-attention (SSA), and an Entangled Feed-Forward Network (EFFN). After layer normalisation, the input is sent to FSA and SSA in parallel; their outputs are fused, renormalised, and fed to EFFN.

FSA captures global frequency dependencies. The input $\hat{P}$ is linearly projected, transformed with FFT, and produces complex-valued queries $Q_{f}$, keys $K_{f}$, and values $V_{f}$. The resulting attention map $\Lambda_{f}$ is split into real and imaginary parts, each soft-maxed and recombined, enabling the model to correlate periodic or textured patterns across the spectrum before transformation back to the spatial domain via IFFT.

SSA models local spatial context efficiently. We employ multi-scale depthwise separable convolution (MSC) to generate $Q_{s}$, $K_{s}$, and $V_{s}$, focusing attention on geometry and local relations.

EFFN performs two-stage cross-domain exchange. First, the fused features are projected separately to spatial and frequency domains. Second, the two outputs are concatenated and re-entered into parallel branches for deeper interaction, forcing repeated alignment between complementary cues. When spatial details are obscured, frequency patterns such as texture still guide discrimination, yielding robust representations with negligible parameter overhead. Parallel FSA and SSA also exploit GPU concurrency for faster inference.

#### 3.2.2. S2-Enhanced CNN-Based Cross-Scale Feature Fusion

Severe scale variation, especially tiny targets buried by repeated down-sampling, motivates the S2-Enhanced CNN-based Cross-Scale Feature Fusion (S2-CCFF). Using a bidirectional topology, it exchanges information among backbone outputs $\left\{S_{2},S_{3},S_{4},S_{5}\right\}$. A top-down semantic path up-samples S5 to S4, concatenates with $S_{4}$, and aggregates them with a RepC3 block, iterating to S2. The fused high-resolution layer $F_{2}$ is then the source of a bottom-up localisation path. After a stride-3 convolution, $F_{2}$ is down-sampled, fused with the semantic output at S3, and processed again by RepC3, propagating precise edges upward while semantic cues flow downward. Thus, each scale receives both rich semantics and fine localisation, boosting small-object detection.

**Figure 4:**
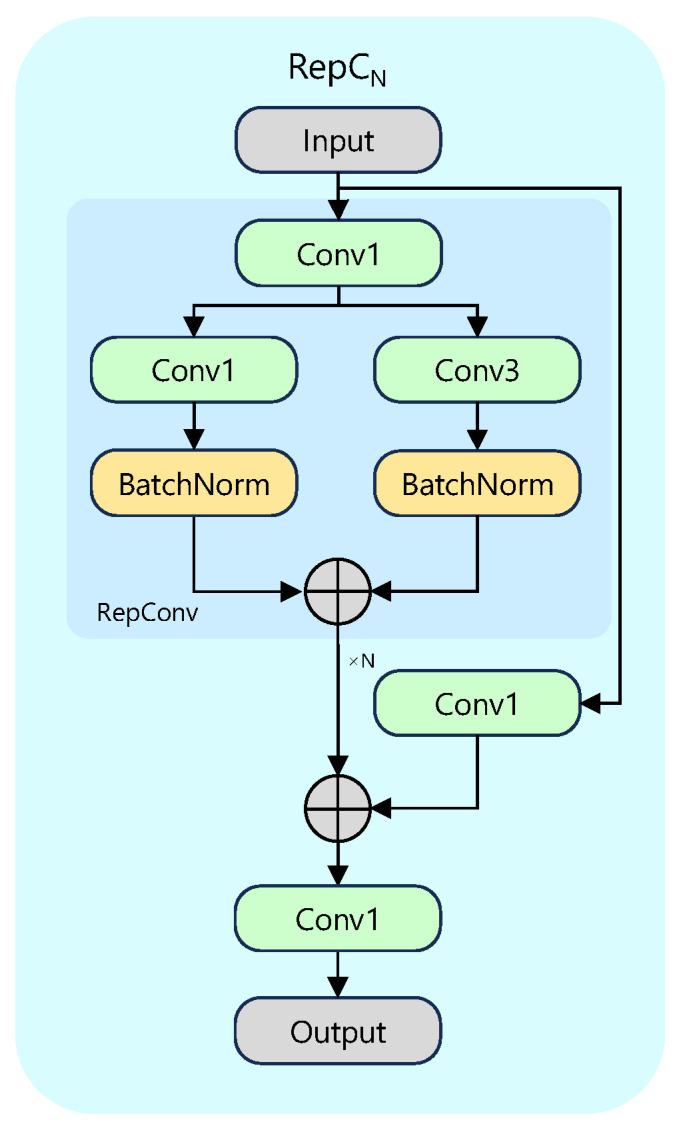
Internal design of the RepC_N_ (*N* = 3) block.

RepC3 is chosen as the fusion unit, see Figure 4. A fixed receptive field limits ordinary convolutions, whereas RepC3 leverages structural re-parameterisation [25]. During training, its RepConv branch mates a 3×3, a 1×1, and an identity pathway, akin to an ensemble of receptive fields. At deployment, these branches are algebraically folded into a single 3×3 convolution by padding the 1×1 kernel and merging batch-normalisation statistics, giving standard 3×3 cost yet richer representation.

### 3.3. Decoder

To remain efficient and free of non-maximum suppression, the decoder adopts the query selection strategy from the baseline RT-DETR [4]. The mechanism, referred to as uncertainty-minimal query selection (UmQS), is designed to provide high-quality initial queries to the decoder. It operates by first using an auxiliary prediction head on the encoder’s output features to generate initial classification scores. Then, the features corresponding to the top-K highest scores are selected to serve as the initial object queries for the decoder layers. This approach ensures that the decoder focuses its attention on feature map regions with a high probability of containing objects, thereby improving both convergence speed and overall efficiency.

These selected queries pass through several identical decoder layers, each applying self-attention to suppress redundancy and cross-attention to gather evidence from encoder features. Training is supervised by a set-to-set loss with Hungarian matching between *N* predictions and *M* ground truths:

(2) $$L_{\mathrm{decoder}}=\lambda_{\mathrm{cls}}L_{\mathrm{cls}}\left(c,\hat{c}\right)+\lambda_{L1}L_{L1}\left(b,\hat{b}\right)+\lambda_{\mathrm{GIoU}}L_{\mathrm{GIoU}}\left(b,\hat{b}\right)$$

where $L_{\mathrm{cls}}$ is the focal loss [26] to tackle class imbalance, and $L_{L1}$ and $L_{\mathrm{GIoU}}$ [27] supervise bounding box regression. Losses are applied to every decoder layer and summed, realising an end-to-end pipeline without postprocessing.

## 4. Experiments and Results

### 4.1. Experimental Setup

#### 4.1.1. Datasets

**Figure 5:**
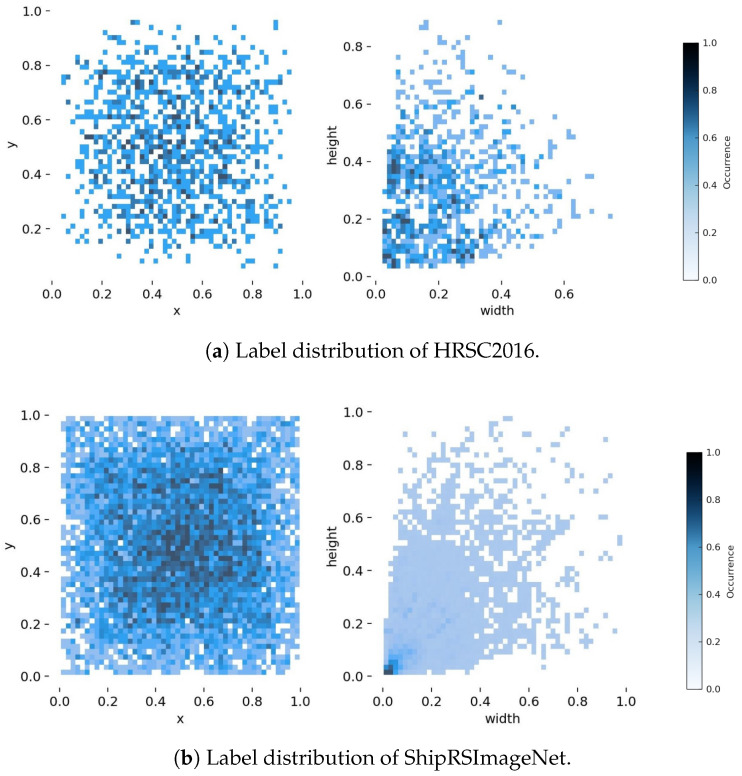
Label distributions of HRSC2016 and ShipRSImageNet. (**Left**): Normalised target centre coordinates (x,y). (**Right**): Normalised target sizes (width, height).

To conduct a comprehensive and multi-faceted evaluation of the proposed MSRS-DETR model, we selected two representative public benchmarks for remote sensing ship detection, namely, HRSC2016 and ShipRSImageNet. These datasets span scenarios from simple to highly complex, enabling an effective assessment of the model’s accuracy, robustness, and generalisation, especially for multi-scale and small-object detection. Figure 5 visualises the differences in target scale and spatial distribution between the two datasets.

HRSC2016 [28] is a classic benchmark for ship recognition in high-resolution optical remote sensing imagery. It contains 1061 images collected from six major ports, officially split into 436 training, 181 validation, and 444 test images, with 2976 annotated ship instances in total. Resolutions range from 0.4 m to 2.0 m and image sizes vary from 300×300 to 1500×900 pixels. Images cover open-sea scenes with simple backgrounds and inshore-port scenes with highly cluttered backgrounds, thereby testing detection performance under diverse conditions. As shown in Figure 5a, the target-size distribution is relatively uniform, making HRSC2016 suitable for evaluating multi-scale detection. Imagery is derived from Google Earth across six harbours; the dataset records harbor, data source, image date, geographic coordinates, resolution layer, and scale.

ShipRSImageNet [29] is larger, more fine-grained, and more diverse. It comprises 3435 images with 17,573 ship instances sourced from various remote sensing platforms worldwide. Compared with HRSC2016, its target sizes vary more widely and include many small objects (Figure 5b). Complex backgrounds and heterogeneous scenes pose additional challenges for feature representation. Consequently, ShipRSImageNet is used in ablation studies to verify the effectiveness and generalisation of each proposed module for small-object detection in complex scenes. Images are collected from multiple sources including xView (WorldView-3, 0.3 m GSD, chips of 930×930), HRSC2016 and FGSD, the Airbus Ship Detection Challenge, and Chinese satellites such as GaoFen-2 and JiLin-1; the official split is 2198/550/687 (train/validation/test).

#### 4.1.2. Evaluation Metrics

Model performance is assessed with a set of accuracy and efficiency metrics. Accuracy follows the COCO protocol and reports precision (P), recall (R), and mean Average Precision (mAP). Efficiency focuses on model complexity and inference speed, quantified by the number of parameters (Params) and theoretical computation (GFLOPs). Definitions are summarised in Table 1. For both HRSC2016 and ShipRSImageNet, detections are evaluated using horizontal bounding boxes (HBB) parameterised as (x,y,w,h), with standard axis-aligned IoU. Unless otherwise noted, we report AP_50_, AP_75_, and mAP_50:95_.

#### 4.1.3. Implementation Details

All experiments were run on a single GeForce RTX 4090 (NVIDIA, Santa Clara, USA) to ensure fair comparison. The software stack consisted of Ubuntu 22.04, PyTorch 2.4.0, CUDA 12.4, and Python 3.12.10. Training employed the AdamW optimiser. Input images were resized to 640×640, the batch size was 8, and training lasted 500 epochs with a cosine-annealing learning-rate schedule. Inference speed (FPS) was measured on single images (batch size = 1) and averaged over the entire test set; preprocessing was included. Key settings were as follows. We trained for 500 epochs with a batch size of 8 at an input size of 640×640. We used AdamW with an initial learning rate of $1\times 10^{-4}$, momentum of 0.9, and weight decay of $1\times 10^{-4}$. Data augmentation applied horizontal flipping with probability 0.5 together with random translation of 0.1 and random scaling of 0.5. We did not use rotation, mosaic, mixup, or copy–paste, and automatic mixed precision was off. We used an IoU threshold of 0.7 and limited the number of detections to 300. We fixed the random seed to 0, enabled deterministic training, and used four data-loading workers.

### 4.2. Comparative Experiments

To validate the overall capability of MSRS-DETR, we conducted a horizontal comparison on the HRSC2016 dataset against several mainstream detectors, including our baseline model, RT-DETR-R18. The experimental results are presented in Table 2.

The experimental results demonstrate that our proposed MSRS-DETR strikes an exceptional balance between accuracy, efficiency, and model complexity, yielding significant performance gains. Compared to the baseline RT-DETR-R18, MSRS-DETR achieves a higher core mAP_50-95_ score along with improvements in mAP_50_ and mAP_75_, all with a remarkable 29.1% reduction in parameters. This result showcases a significant performance boost alongside model lightweighting. It is noteworthy that while the theoretical computation (GFLOPs) of MSRS-DETR is higher than the baseline, our subsequent ablation study shows that its practical inference speed is superior. When compared to YOLOv8m, which has similar computational load, MSRS-DETR achieves a 0.18% (HRSC2016) and 0.28% (ShipRSImageNet) higher mAP_50-95_ with fewer GFLOPs. Compared to YOLOv8s, which has a similar parameter count, MSRS-DETR surpasses it by 2.59% (HRSC2016) and 4.92% (ShipRSImageNet) in the core mAP_50-95_ metric. Furthermore, when measured against the top-performing RT-DETR-R101, MSRS-DETR uses only 18.9% of its parameters and 29.8% of its GFLOPs while lagging by a mere 0.55% in mAP_50-95_ on HRSC2016 and exceeding it by 1.38% on ShipRSImageNet. Evidently, MSRS-DETR maintains high accuracy while significantly reducing model complexity and computational cost, demonstrating its outstanding performance on multi-scale ship remote sensing object detection tasks.

### 4.3. Ablation Study

To clearly validate the individual effectiveness and synergistic effects of the proposed C2f_FAT_NET backbone, ETB module, and S2-CCFF module, we conducted a series of ablation experiments. Starting with RT-DETR-R18 as the baseline, we progressively integrated our proposed components and evaluated the performance on both the HRSC2016 and ShipRSImageNet datasets. The results are detailed in Table 3.

The results of the ablation study show that isolating the introduction of the C2f_FAT_NET backbone reduces the model’s parameter count from 19.9 M to 15.2 M (a 23.6% decrease) and lowers GFLOPs from 56.9 G to 51.3 G. While making the model more lightweight, the core mAP_50-95_ metric improved by 0.98% on HRSC2016 and 0.34% on ShipRSImageNet. This confirms that the FATBlock-enhanced C2f_FAT_NET, with its powerful frequency-domain feature extraction, can achieve more efficient multi-scale feature extraction in RSI with fewer parameters and lower computational cost, thus achieving performance gains alongside backbone lightweighting.

When we introduced the ETB module for feature extraction at the top of the encoder, the core mAP_50-95_ metric improved by 0.67% and 0.34% on the two datasets, respectively, with almost no change in parameters or GFLOPs. Notably, the practical inference speed (FPS) increased by a remarkable 52.1% on HRSC2016 and 40.2% on ShipRSImageNet. This provides strong evidence that ETB’s unique frequency–spatial entanglement mechanism can capture more discriminative deep features, and its highly parallel structure effectively utilises the computational power of modern GPUs, leading to a substantial boost in practical inference efficiency without increasing the theoretical computational burden.

The isolated introduction of the S2-CCFF module yields the most significant accuracy gains on the more challenging ShipRSImageNet dataset, which is rich in small targets, with an improvement of 2.13% in mAP_50-95_. Although this module increases GFLOPs due to processing a higher-resolution feature map, the resulting accuracy benefit is substantial. This demonstrates that injecting the high-resolution details from the S2 layer into the bidirectional fusion path is an effective strategy for preserving and utilising key small-object cues and reducing the missed detection rate.

The full MSRS-DETR model, combining all three modules, achieved the best mAP_50-95_ performance across all configurations on both datasets, improving upon the baseline by 1.69% and 2.16%, respectively. The final performance gain surpasses that of any partial combination, indicating a strong synergistic effect among our designed components. C2f_FAT_NET provides richer features at the source, the ETB module refines deep semantics, and the S2-CCFF module ensures effective detail fusion, together forming an efficient and powerful feature processing pipeline. With a 29.1% reduction in parameters compared to the baseline, the MSRS-DETR model achieves higher detection accuracy and faster inference speed (FPS increased by 12.4% and 8.4% on the two datasets), striking an optimal balance between model complexity, inference efficiency, and detection performance. While the ETB’s parallel feature extraction demonstrably improves runtime and accuracy in isolation (Table 3), the end-to-end speedup of the full model is constrained by the S2-CCFF detail-extraction and fusion strategy on the high-resolution path; consequently, overall throughput improves only modestly over the baseline but yields the best accuracy–efficiency trade-off.

### 4.4. Visual Results

**Figure 6:**
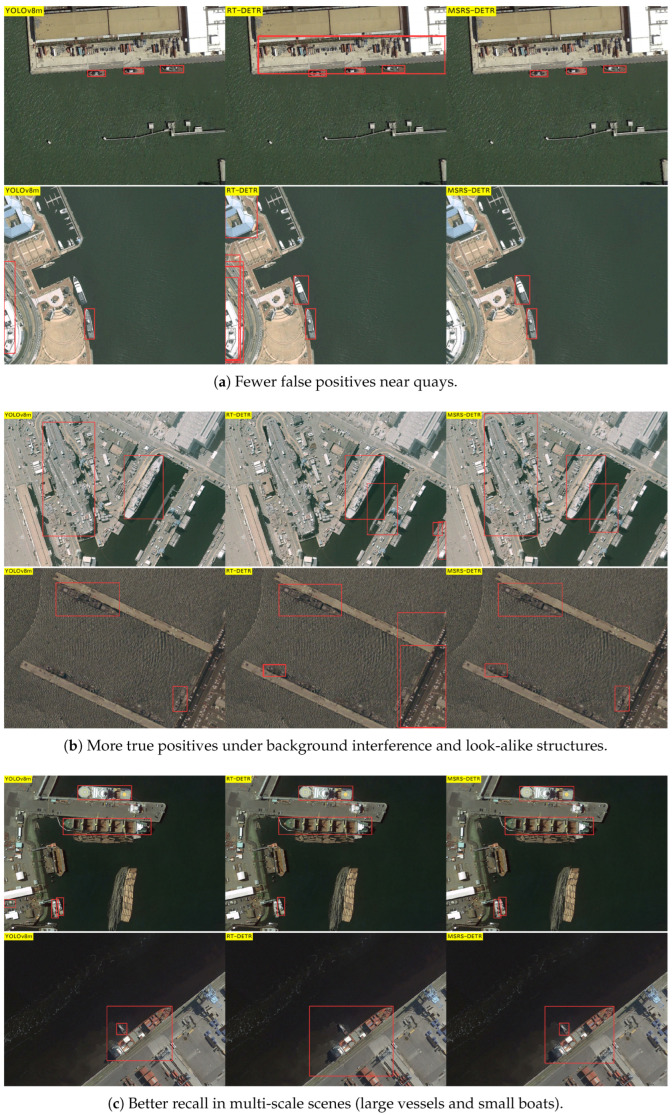
Qualitative comparison on HRSC2016. Each triptych shows YOLOv8m (**left**), RT-DETR (**middle**), and MSRS-DETR (**right**). MSRS-DETR reduces false positives and improves recall under clutter and strong scale variation.

**Figure 7:**
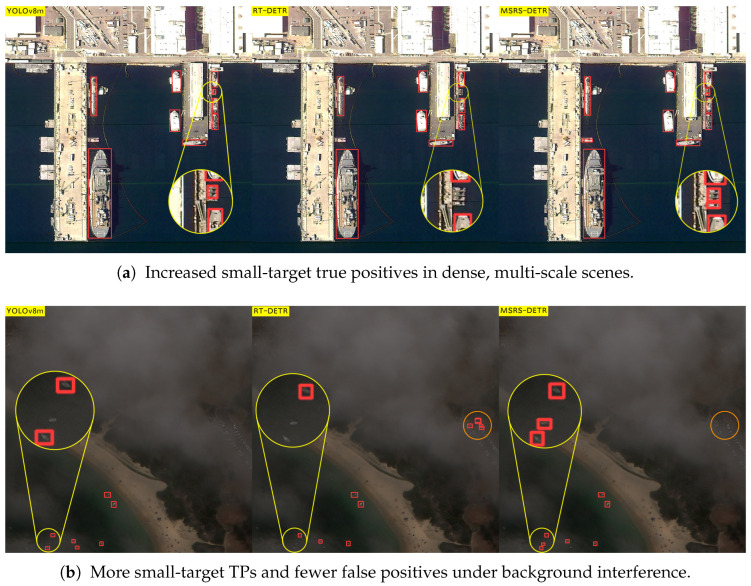
Qualitative comparison on ShipRSImageNet. Each triptych shows YOLOv8m (**left**), RT-DETR (**middle**), and MSRS-DETR (**right**). Our method better localises dense small vessels and suppresses background-induced false alarms.

We further performed qualitative analyses to visualise improvements in challenging scenarios. Figure 6 compares MSRS-DETR with the baseline on HRSC2016, illustrating reductions in false positives, increased true positives in cluttered backgrounds, and superior multi-scale detection. Concretely, Figure 6a shows that MSRS-DETR yields fewer onshore false positives (e.g., quayside structures), Figure 6b demonstrates more reliable recognition under background interference and look-alike structures where the baselines exhibit both false alarms and misses, and Figure 6c highlights better recall of small objects in multi-scale scenes. Figure 7 shows similar advantages on ShipRSImageNet, particularly for dense small targets, evidencing the efficacy of S2-CCFF. Specifically, in Figure 7a YOLOv8m exhibits imprecise delineation of small targets and RT-DETR shows higher miss rates, whereas MSRS-DETR localises dense, multi-scale vessels more accurately; in Figure 7b, under background interference, MSRS-DETR achieves more true positives with fewer false positives than the baselines. These visual results corroborate the quantitative findings and highlight the balanced accuracy, speed, and efficiency achieved by MSRS-DETR.

### 4.5. Discussion

MSRS-DETR improves detection in cluttered ports and dense small-object scenes by aligning frequency-aware feature extraction with an NMS-free DETR pipeline. The gains follow the roles of the three components and the patterns in Section 4.4. C2f_FAT_NET strengthens fine-grained cues at the source and stabilises optimisation under heterogeneous backgrounds, which supports higher recall and fewer background-induced false alarms. ETB couples frequency and spatial information and reduces spectral bias, which preserves high-frequency detail that characterises small vessels and textured surroundings and also explains the strong runtime gains when ETB is used alone. S2-CCFF injects shallow high-resolution signals and propagates edges upward while semantics flow downward, which increases small-object recall in crowded regions. This benefit introduces extra work along the fusion path and bounds the end-to-end speedup once all modules are assembled. The trade-off is consistent with the ablation in Table 3 and with the qualitative panels that show fewer quayside false positives and better localisation of small boats under interference.

The component-to-failure-mode mapping is clear in practice. Extremely low-contrast targets can still be missed when wakes or shadows dominate the local spectrum, and S2-CCFF reduces this miss rate by recovering edges while ETB sharpens texture cues. Look-alike human-made structures can trigger false alarms, and C2f_FAT_NET improves class separation through more selective early features while ETB suppresses background leakage. Very large targets can be partially detected when effective context is limited, and the multi-scale route mitigates this behaviour by passing both localisation detail and deep semantics across scales. Together, these mechanisms produce the accuracy and efficiency balance reported for the full model.

The improvements we report come from the frequency–spatial fusion architecture that operates upstream of the box head and is therefore representation agnostic. To isolate architectural effects and preserve internal validity with the RT DETR baseline, we adopt horizontal bounding boxes, which keep Hungarian matching, losses, and uncertainty minimal query selection unchanged. Introducing oriented boxes would add angle parameterisation, periodicity handling, and rotated IoU and assignment, which could confound attribution of gains to our modules. HRSC2016 and ShipRSImageNet both natively support horizontal annotations and evaluation, which allows stable and directly comparable reporting without redesigning protocols. In future work we will add oriented detection and oriented metrics to complete the picture and to compare against methods that use rotated heads.

MSRS-DETR is positioned in the real-time end-to-end line that begins with DETR set prediction without NMS and extends to RT DETR for throughput. Our pathway differs from common FPN or EBiFPN necks that fuse only in the spatial domain. Frequency analysis appears in the backbone through FAT and in the encoder through ETB, so the fusion module receives inputs whose fine detail is already enhanced rather than recovered post hoc. The shallow bidirectional fusion is instantiated by RepC_N_ with N=3 in our design and targets small-object detail while leaving the decoder unchanged in the RT DETR sense. This division of labour helps explain why accuracy improves most on ShipRSImageNet, where tiny targets dominate, while the end-to-end property is preserved.

The framework is practical with public cloud-hosted imagery and can run economically at scale. A typical workflow accesses Sentinel 2 L2A and Landsat ARD in place, applies quality masks such as the SCL layer for Sentinel 2 and QA bits for Landsat, normalises radiometry when mixed sensors are used, and windows space and time to produce chips at the detector input size. This pre-detector adaptation layer documents provenance and explains tolerance to varied spatial resolutions, and the detector then serves as the core of a multi-scale pipeline on cloud-native platforms with object storage and on-the-fly tiling and masking. We expect these steps to generalise beyond ships and to broaden usage in large-area monitoring where repeated coverage and low cost are important.

## 5. Conclusions

To overcome the performance bottleneck caused by the exclusive use of spatial information in existing remote sensing detectors, we have presented MSRS-DETR, a paradigm that deeply fuses spatial and frequency cues. The proposed model integrates a frequency-aware backbone network C2f_FAT_NET, an Entanglement Transformer Block (ETB), and a shallow-feature-enhanced bidirectional fusion framework S2-CCFF into the backbone, encoder, and fusion stages, respectively. This design systematically alleviates small-object information loss and background interference from the initial feature extraction stage through multi-scale feature fusion.

Experiments on the HRSC2016 and ShipRSImageNet benchmarks show that MSRS-DETR markedly reduces parameter count while simultaneously improving detection accuracy and inference speed, confirming the effectiveness of joint spatial–frequency fusion in complex remote sensing scenarios for multi-scale ship remote sensing object detection. While the proposed method shows strong performance on these ship benchmarks, future work should focus on validating this approach on a wider variety of object classes (e.g., vehicles, aircraft) to rigorously assess its generalisability. This balanced outcome is achieved through the synergy of its components: the C2f_FAT_NET backbone reduces parameters while enriching features, the ETB block significantly boosts practical inference speed, and the S2-CCFF module trades some of that speed gain for critical accuracy improvements on small targets. Future work will explore lighter and more adaptive spatial–frequency co-learning strategies, aiming to apply the dual-domain feature extraction and fusion scheme more efficiently to even more complex, multi-scenario, and multi-scale remote sensing detection tasks.

## Figures and Tables

**Table 1:** Definitions of evaluation metrics. TP, FP, and FN denote true positives, false positives, and false negatives, respectively. $N_{\mathrm{cls}}$ is the number of classes.

Category	Metric	Definition
Accuracy	*P*	$P=\frac{\mathrm{TP}}{\mathrm{TP}+\mathrm{FP}}$
	*R*	$R=\frac{\mathrm{TP}}{\mathrm{TP}+\mathrm{FN}}$
	AP	$\mathrm{AP}=\int_{0}^{1}P\left(R\right)\;dR$
	mAP	$\mathrm{mAP}=\frac{1}{N_{\mathrm{cls}}}\sum_{i=1}^{N_{\mathrm{cls}}}{\mathrm{AP}}_{i}$
	mAP_50_	mAP at IoU = 0.50
	mAP_75_	mAP at IoU = 0.75
	mAP_50-95_	Average of mAP at IoU = 0.50:0.05:0.95
Efficiency	Params (M)	Trainable parameters in millions
	GFLOPs	Giga floating-point operations

**Table 2:** Performance comparison on HRSC2016 (H)/ShipRSImageNet (S). All metrics in each cell are reported as H/S. **Bold** indicates the best per column; underline indicates the second best.

Model	Params (M)	GFLOPs	mAP_50_	mAP_75_	mAP_50-95_
YOLOv8s	**11.1**	**28.4**	93.33/80.05	91.04/72.23	83.62/62.88
RTDETR-HGBlock	32.0	103.4	92.99/84.12	90.79/75.60	84.47/66.07
RTDETR-R18	19.9	56.9	92.93/84.09	90.58/74.84	84.52/65.64
YOLOv8m	25.8	78.7	93.62/84.36	91.95/77.27	86.03/67.52
RTDETR-R101	74.7	247.1	**94.33**/83.90	**92.21**/75.89	**86.76**/66.42
MSRS-DETR	14.1	73.7	94.06/**85.09**	92.08/**78.14**	86.21/**67.80**

**Table 3:** Ablation study of the proposed modules. All metrics are presented in the format of HRSC2016 (H)/ShipRSImageNet (S). The checkmark (✓) indicates the module is included. The final model is highlighted in **bold**.

Baseline	C2f_FAT_NET	ETB	S2-CCFF	Params (M)	GFLOPs	mAP_50_	mAP_75_	mAP_50-95_	FPS
✓				19.9	56.9	92.93/84.09	90.58/74.84	84.52/65.64	169/179
✓	✓			15.2	51.3	93.86/84.28	92.00/75.50	85.50/65.98	157/154
✓		✓		20.0	57.3	94.05/83.59	91.48/75.16	85.19/65.98	257/251
✓			✓	18.6	78.1	93.03/85.35	90.45/78.20	84.85/67.73	161/151
✓	✓	✓		15.4	51.6	94.45/84.25	92.18/75.31	86.20/65.25	195/232
✓	✓		✓	14.0	73.3	92.81/84.91	90.57/76.97	84.76/66.77	162/166
✓		✓	✓	18.7	78.5	93.89/84.93	92.03/77.79	85.33/67.59	203/213
✓	✓	✓	✓	**14.1**	**73.7**	**94.06**/**85.09**	**92.08**/**78.14**	**86.21**/**67.80**	**190**/**194**

## Data Availability

The HRSC2016 and ShipRSImageNet datasets used in this study are publicly available resources. The trained model weights have been made publicly available on GitHub (https://github.com/loges00/MSRS-DETR/releases/tag/weights, accessed on 25 August 2025).
